# Impact of Physiotherapy Based on the Rigo Concept and Whole-Body Vibration on Sagittal Spinal Curvatures, Trunk Symmetry, and the Angle of Trunk Rotation in Adolescents with Idiopathic Scoliosis

**DOI:** 10.3390/jcm15041386

**Published:** 2026-02-10

**Authors:** Paulina Ewertowska, Marta Flis, Joanna Kujałowicz, Borislav Chongov, Dariusz Czaprowski

**Affiliations:** 1 Department of Physical Culture, Gdansk University of Physical Education and Sport, 80-336 Gdansk, Poland; marta.flis@awf.gda.pl; 2 Korrektiv, 80-460 Gdansk, Poland; joanna.kujalowicz@gmail.com; 3 Department of TM of the Kinesiotherapy, National Sports Academy “Vassil Levski”, 1700 Sofia, Bulgaria; bobychongov@gmail.com; 4 Specialized Orthopedic University Hospital “Prof. B. Boichev”, 1614 Sofia, Bulgaria; 5 Department of Physiotherapy, School of Public Health, Collegium Medicum, University of Warmia and Mazury, 10-720 Olsztyn, Poland; dariusz.czaprowski@interia.pl; 6 Center of Body Posture, 10-054 Olsztyn, Poland

**Keywords:** spinal deformity, specific physiotherapy, Barcelona Scoliosis Physical Therapy School, vibrating platform

## Abstract

**Background:** Conservative treatment for adolescent idiopathic scoliosis (AIS) includes physiotherapeutic scoliosis-specific exercises (PSSE) and bracing. One PSSE-based approach is the Rigo Concept, which emphasizes three-dimensional (3D) postural correction, expansion techniques, muscle activation, and postural integration. Recently, increasing interest has been directed toward incorporating whole-body vibration (WBV) into physiotherapy. WBV is a reflex-based neuromuscular training method shown to improve muscle strength and power and enhance proprioception, which may be beneficial in the treatment of AIS. **Objectives**: This study aimed to assess the effects of physiotherapy based on the Rigo Concept combined with WBV on sagittal spinal curvatures, trunk symmetry, and the angle of trunk rotation (ATR) in girls with AIS. **Methods**: This prospective controlled experimental study included 45 girls (12.8 ± 1.7 years) with AIS who participated in a 5-day physiotherapy session based on the Rigo Concept. Of these, 22 participants additionally received WBV using a Galileo Med 35 platform (3 × 3 min/day, frequency 25 Hz, peak-to-peak displacement 2 mm), forming the Rigo–WBV group. The remaining participants received the Rigo Concept alone (Rigo–ONLY). Participants were allocated to the study groups using a quasi-random method based on the order of enrollment. ATR was defined as the primary endpoint, while thoracic kyphosis, lumbar lordosis, sacral slope, coronal balance, and scapular position were considered secondary outcomes. All outcomes were assessed before and after the intervention. **Results**: Neither the Rigo–WBV nor the Rigo–ONLY intervention affected sagittal spinal curvatures (*p* > 0.05). Coronal balance improved in both the Rigo–WBV (Δ 0.5 cm, *p* < 0.001) and Rigo–ONLY groups (Δ 0.4 cm, *p* = 0.005). In the Rigo–ONLY group, an improvement in scapular height asymmetry was observed (Δ 1.1°, *p* = 0.010). Following the Rigo–WBV intervention, ATR decreased in the main thoracic (Δ 1.9°, *p* < 0.001), thoracolumbar (Δ 1.9°, *p* < 0.001), lumbar curve (Δ 2.1°, *p* < 0.001), and pelvis (Δ 1.0°, *p* < 0.001). In the Rigo–ONLY group, a reduction in ATR was observed only in the thoracolumbar curve (Δ 1.9°, *p* < 0.001). **Conclusions**: In terms of clinical and postural changes, five-day physiotherapy based on the Rigo Concept, with or without WBV, does not influence sagittal spinal curvatures in girls with AIS. Both interventions may improve coronal balance. Moreover, the Rigo Concept combined with WBV may reduce ATR.

## 1. Introduction

Adolescent idiopathic scoliosis (AIS) is a structural three-dimensional (3D) spinal deformity characterized by lateral deviation and a Cobb angle greater than 10°. It affects children aged 10–16 years, with a higher risk of curve progression observed in females [1,2]. The prevalence of AIS ranges from 2 to 4%. Its aetiology remains unknown, although it is likely that patients with AIS present several abnormal growth-related factors [1]. If left untreated, AIS with a Cobb angle exceeding 30°may continue to progress after skeletal maturity, potentially leading to back pain, pulmonary dysfunction, aesthetic concerns, self-image issues, functional impairments, and balance disturbances [2], all of which negatively affect quality of life [1,2]. Treatment of AIS includes conservative and surgical management, depending on the Cobb angle and the risk of progression [1]. According to the Society on Scoliosis Orthopaedic and Rehabilitation Treatment (SOSORT), physiotherapeutic scoliosis-specific exercises (PSSE) are recommended as the first-line treatment for mild curves and as an adjunct to bracing [1,2]. PSSE should incorporate 3D self-correction, training in activities of daily living, and stabilisation of the corrected posture [2], with the aim of improving aesthetic appearance [3]. The Rigo Concept is based on SOSORT guidelines and integrates a classification system for both PSSE and bracing. Its therapeutic goals include improving aesthetics, preventing curve progression, increasing patient education and activity, enhancing breathing function, promoting functional mobility and daily activities, and improving self-esteem, self-image, and pain levels [4]. Although studies have reported positive effects of PSSE—such as improvements in Cobb angle [2,4,5,6], trunk symmetry [3], angle of trunk rotation (ATR) [2,4,5,6,7], balance [1,5], and quality of life [2]—there are still relatively few high-quality studies [3] clearly indicating the effectiveness of the Rigo Concept, as well as other methods used in the physiotherapy of scoliosis. Available studies are non-randomized, involve heterogeneous intervention protocols, and are not conducted in representative populations of children with scoliosis. Furthermore, a review of the current literature did not identify studies demonstrating improvements in clinical parameters such as sagittal spinal curvatures, coronal balance, or ATR specifically attributable to the application of the Rigo Concept. For these reasons, the effectiveness of PSSE remains a subject of ongoing debate among specialists involved in scoliosis management. This limitation is partly due to the methodological challenges associated with applying a uniform intervention over a long period of time in order to observe long-term effects of physiotherapy [8], which aims to improve both clinical and radiological outcomes.

Recently, increasing interest has been observed in incorporating vibration platforms into physiotherapy. Whole-body vibration (WBV) is a reflex-based and safe form of neuromuscular training [9,10]. It is an effective method for enhancing neuromuscular activation and proprioceptive feedback through various vibration frequencies and amplitudes, and it has been shown to improve muscle strength, power, balance, and proprioception [9,10,11]. Consequently, WBV is increasingly used in physiotherapy for neurological conditions such as Parkinson’s disease, multiple sclerosis, stroke, and cerebral palsy, as well as in athletic training to enhance performance [11]. In the available literature, only one study investigating the use of WBV in scoliosis therapy has been identified [9]. According to the manufacturer, low frequencies (5–10 Hz) are used for balance and mobilization, particularly in neurological populations, medium frequencies (12–20 Hz) stimulate muscle function, and high frequencies (25–40 Hz are applied to increase muscle power and endurance [10,12]. To date, evidence regarding the use of WBV in scoliosis is limited to a single study reporting effects on the Cobb angle [9]. Therefore, the present study aims to explore the potential added value of incorporating WBV into Rigo Concept-based physiotherapy by examining short-term clinical and postural outcomes, such as ATR and coronal balance, using non-radiographic assessment methods. According to the Rigo Concept, 3D passive spinal correction should be combined with muscle activation during exercise [4]. This activation is intended to initiate and maintain spinal correction. Available evidence suggests that high-frequency WBV may enhance muscle function, thereby increasing the corrective effect on the spine in all three planes [9,11]. Based on these findings, we hypothesized that combining the Rigo Concept with WBV could have a beneficial effect on conservative AIS treatment by stimulating proprioception and activating stabilizing muscles during correction in the standing position.

The aim of this study was to analyse the effects of physiotherapy based on the Rigo Concept combined with WBV on increasing sagittal spinal curvatures, improving trunk symmetry in the frontal plane, and reducing ATR in girls with AIS.

## 2. Materials and Methods

### 2.1. Recruitment of Participants

Children and their parents participated in the study voluntarily. The inclusion criteria were female sex, age from 10 to 16 years, and a diagnosis of AIS. Parents were required to provide physician approval, complete medical documentation, and up-to-date radiographic examinations (X-rays). All participants had a history of physiotherapy delivered using various therapeutic approaches. The initial phase of the study included an interview covering basic information, potential contraindications to testing, and anthropometric measurements. Body height in the standing position was measured using an HM 202P stadiometer (Medwil, Strażów, Poland), and body mass was assessed using an InBody720 device (InBody USA, Cerritos, CA, USA) [13]. Radiological parameters, including the Cobb angle and Risser test, were assessed by a physiotherapist (researcher 1; R1) with 10 years of experience in scoliosis management.

**Figure 1:**
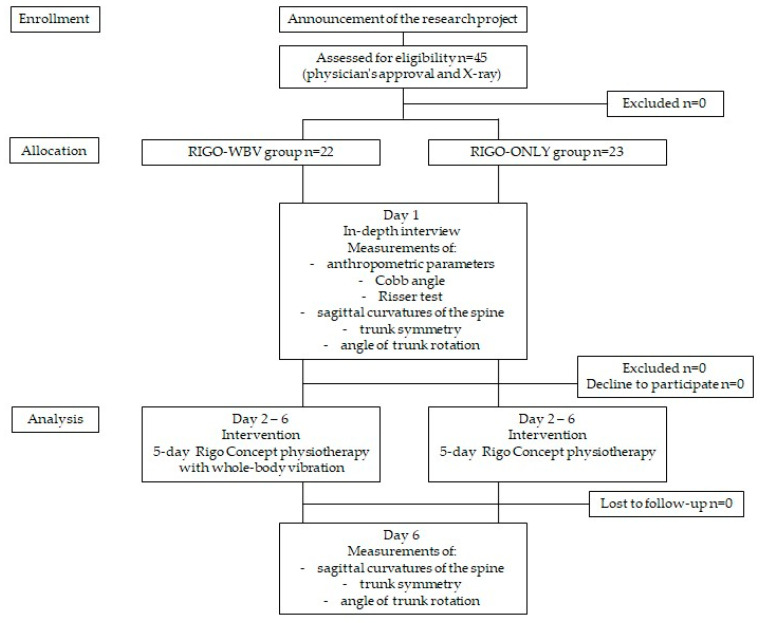
The recruitment process for the participants.

Participants with contraindications to training on a vibration platform were excluded. These contraindications included thrombosis, body implants, musculoskeletal inflammation, arthropathy, tendonitis, hernia, disc herniation, recent fractures, kidney stones, recent scars, recent surgery, rheumatoid arthritis, neuropathy, epilepsy, or discomfort or malaise experienced during vibration exposure. Additional exclusion criteria included previous spinal surgery, as well as injuries or back pain or lower limb pain within the preceding six months [12]. The recruitment process is presented in Figure 1.

### 2.2. Participants’ Baseline Measurements

Ultimately, 45 girls with AIS (age 12.8 ± 1.7 years) participated in the study. Participants were assigned to one of two groups: physiotherapy sessions based on the Rigo Concept combined with WBV (*n* = 22; Rigo–WBV) or physiotherapy sessions based on the Rigo Concept alone (*n* = 23; Rigo–ONLY) (Table 1). Participants were allocated to the study groups using a quasi-random method based on the order of enrollment by R1. There were no statistically significant differences between the groups in baseline parameters (*p* > 0.05).

Prior to the main part of this study, the total number of scoliotic curves was determined, and the Cobb angle was measured. All participants presented with mild or moderate AIS (Table 2), and no significant differences between groups were observed (*p* > 0.05). Due to the small number of proximal thoracic curves, this curve type was excluded from further analysis.

At baseline, there were no significant differences between the Rigo–WBV and Rigo–ONLY groups with respect to thoracic kyphosis (*p* = 0.101), upper thoracic kyphosis (*p* = 0.704), lower thoracic kyphosis (*p* = 0.271), lumbar lordosis (*p* = 0.800), or sacral slope (*p* = 0.549). In the frontal plane, no significant differences were found between the Rigo–WBV and Rigo–ONLY groups regarding coronal balance (*p* = 0.597) or the difference in the distances of the scapulae from the spine (*p* = 0.687). However, scapular height asymmetry was significantly greater in the Rigo–ONLY group (*p* = 0.035). In addition, there were no significant differences between the Rigo–WBV and Rigo–ONLY groups in ATR for the thoracolumbar curve (*p* = 0.420), lumbar curve (*p* = 0.120), or at the posterior superior iliac spine (PSIS) (*p* = 0.089). However, the Rigo–WBV group demonstrated a significantly higher ATR for the main thoracic curve (*p* = 0.009). The results are presented in Table 3.

### 2.3. Study Design

This study was designed as a controlled experimental trial with two groups and systematic sample selection. Participants were allocated to the study groups using a quasi-random method based on the order of enrollment by R1. The first group underwent physiotherapy sessions based on the Rigo Concept combined with WBV, while the second group received physiotherapy sessions based on the Rigo Concept alone. Both interventions were conducted by Researcher 2 (R2). Sagittal spinal curvatures, parameters in the transversal plane, and trunk symmetry parameters in the frontal plane were assessed before the first day of the physiotherapy sessions and 3 h after completion of the fifth day of physiotherapy by R1. ATR was defined as the primary endpoint, as it represents the most important clinical parameter for monitoring the effectiveness of scoliosis treatment, apart from radiological assessment. Secondary outcomes included thoracic kyphosis, lumbar lordosis, sacral slope, coronal balance, and scapular position. Due to organizational constraints within the clinical setting, R1 was responsible for group allocation and for conducting pre- and post-intervention outcome measurements, while R2 delivered the intervention. As a result, blinding the researcher was not feasible. However, all assessments were performed according to predefined, standardized procedures to minimize potential assessment bias.

The study protocol was approved by the Bioethical Committee of the Gdansk University of Physical Education and Sport (No. 25/01) on 4 April 2025 and was conducted in accordance with the Declaration of Helsinki of the World Medical Association. Written informed consent was obtained from the parents of all participants. All participants were informed about the potential risks and benefits of the study and were free to withdraw at any time. The study was registered as a clinical trial (ClinicalTrial.gov identifier: NCT07285551 on 2 December 2025.

### 2.4. Intervention

**Figure 2:**
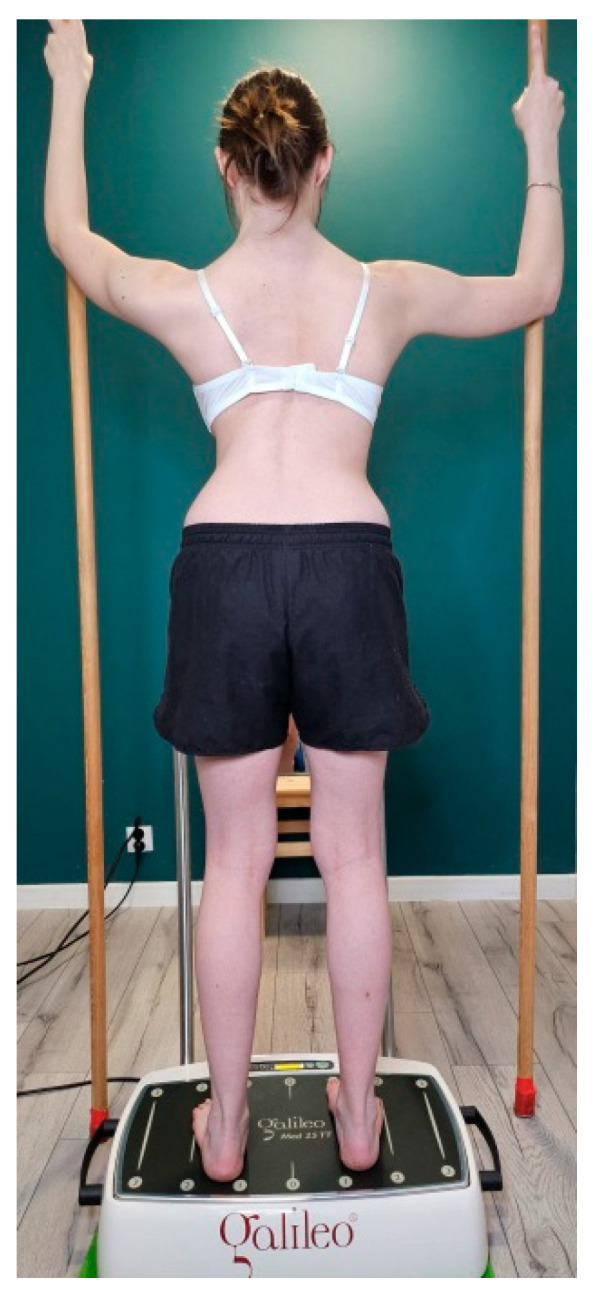
Standing position with two poles during whole-body vibration.

This study was conducted in a physiotherapy clinic specifically adapted and equipped for the implementation of the Rigo Concept. According to the Rigo Concept classification, participants underwent a 5-day physiotherapy session, during which the type of scoliosis was determined, and appropriate exercises were selected by a physiotherapist (R2) with 10 years of experience in scoliosis management and certified in the Rigo Concept approach [3,4]. The exercises followed the four general principles of the Rigo Concept approach: 3D postural correction, expansion technique, muscle activation, and integration [4,8]. Physiotherapy sessions were delivered for 3 h per day (between 9:00 a.m. and 1:00 p.m.), with three 15 min breaks, and were supervised by R2. Additionally, girls in the Rigo–WBV group were exposed once daily to WBV using a side-alternating vibration platform, Galileo Med 35 (Novotec Medical GmbH, Pforzheim, Germany) at a constant frequency of 25 Hz and a peak-to-peak displacement of 2 mm throughout the 5-day physiotherapy session. Each WBV session was performed in a standing position with two poles (Figure 2), was supervised by R2, and consisted of three 3 min vibration bouts, each followed by a 3 min rest period [9,14,15]. During WBV exposure, participants performed an exercise in accordance with the principles of the Rigo Concept, with particular emphasis on elongation and expansion techniques. They were instructed to maintain slight knee flexion to reduce the transmission of vibrations to the head [16]. All participants were familiar with the standing-with-two-poles exercise from the Rigo Concept approach and had prior experience with WBV. During the familiarization session, participants performed exercises on the vibration platform for 10 s. To prevent slipping, the exercise was performed barefoot [9].

### 2.5. Measurements of Sagittal Curvatures

Sagittal spinal curvatures were assessed using a Baseline digital inclinometer (MVS in Motion, Roeselare, Belgium) by R1 [17]. Measurements were performed in a relaxed standing position, with participants wearing underwear and without shoes [17]. The initial assessment included evaluation of the thoracic kyphosis (upper and lower parts), lumbar lordosis, and sacral slope [17,18,19]. The measurement procedure was as follows: (1) thoracic kyphosis—the inclinometer was reset at the thoracolumbar junction (Th12–L1), and the value was recorded at the cervicothoracic junction (C7–Th1); (2) upper thoracic kyphosis—the inclinometer was reset at the C6–C7 junction, and the value was recorded at the C7–Th1 junction; (3) lower thoracic kyphosis—the inclinometer was reset at the thoracolumbar junction (Th12–L1), and the value was recorded at the C6–C7 junction; (4) lumbar lordosis—the inclinometer was reset at the lumbosacral junction (L5–S1), and the value was recorded at the thoracolumbar junction (Th12–L1); (5) sacral slope—the inclinometer was reset in the horizontal position, and the value was recorded at the lumbosacral junction (L5–S1) [17,18]. For analysis, the mean value of three measurements was used [17]. Measurements using the inclinometer are characterized by repeatability [17].

### 2.6. Measurements of Trunk Symmetry

Trunk symmetry parameters assessed in the frontal plane included coronal balance, scapular height asymmetry, and the difference between the distances of the scapulae from the spine. All assessments were conducted by R1. Measurements were performed in a relaxed standing position without shoes. For coronal balance assessment, a plumb line was positioned at the level of the occipital prominence, and the distance between the plumb and the gluteal creft was measured using a rigid millimetre ruler [20]. For the assessment of scapular height asymmetry, the inferior angles of the scapulae were marked bilaterally [21]. A rigid ruler was placed across these landmarks, and a scoliometer was positioned on the ruler to record the angle of inclination [22]. Additionally, the distances from the inferior angles of the scapulae to the line of the spinous processes were measured on both the left and right sides using a ruler, and the difference between sides was calculated [23,24]. Measurements of scapular positioning are characterized by low repeatability [23].

### 2.7. Measurements of Angle of Trunk Rotation

ATR was assessed by R1 during the Adams forward bend test using a Baseline scoliometer (MoVeS, Kraków, Poland) [25,26]. Participants were examined wearing underwear and without shoes. Each participant stood in a relaxed upright position with feet placed hip-width apart and was instructed to place the hands together and slowly bend forward, directing the hands toward the center of support [25,26]. The scoliometer was applied horizontally, and the examiner recorded the magnitude and direction of ATR at the proximal and main thoracic, thoracolumbar, and lumbar curve, as well as at the level of the PSIS. ATR was analyzed separately for individual spinal segments. The number of curves and ATR values included in the analysis corresponded to the number of spinal curvatures identified on frontal-plane radiographs. Spinal segments without a curve were excluded from the comparative analysis. ATR measurements obtained using a scoliometer are characterized by repeatability and reliability [27].

### 2.8. Statistical Analysis

Statistical analysis was performed using Statistica 13.1 (StatSoft, Kraków, Poland). Descriptive statistics and absolute mean changes were calculated. The normality of variable distributions was assessed using the Shapiro–Wilk test. An independent-samples t-test was used to compare variables with a normal distribution between groups. To examine between-group differences over time, a two-way ANOVA with factors group (Rigo–WBV vs. Rigo–ONLY) and time (pre- vs. post-intervention) was applied. When a significant effect was detected, post hoc comparisons were performed using the Tukey test. Effect sizes were calculated and interpreted according to Cohen’s criteria as small (d ≈ 0.2), medium (d ≈ 0.5), and large (d ≥ 0.8). The value α < 0.05 was adopted as the level of significance.

## 3. Results

### 3.1. Results of the Sagittal Curvatures

Results of sagittal curvatures are presented in Table 4. Two-way ANOVA revealed no significant interaction effect (F  =  0.071; *p*  =  0.791), no significant main effect of group (F  =  3.538; *p*  =  0.067), and no significant main effect of time (F  =  3.145; *p*  = 0.084) for thoracic kyphosis.

For upper thoracic kyphosis, no significant interaction effect was found (F = 0.004, *p* = 0.951), and no significant main effect of group was observed (F = 0.153, *p* = 0.698). However, a significant main effect of time was identified (F = 7.585, *p* = 0.009). Post hoc comparisons for the main effect of time did not reveal significant changes following either the Rigo–WBV (*p* = 0.214) or the Rigo–ONLY intervention (*p* = 0.211).

No significant interaction effect (F = 0.014, *p* = 0.905), no significant main effect of group (F = 1.159, *p* = 0.288), and no significant main effect of time (F = 0.992, *p* = 0.325) were observed for lower thoracic kyphosis.

Similarly, no significant interaction effect (F = 0.381, *p* = 0.540), no significant main effect of group (F = 0.243, *p* = 0.624), and no significant main effect of time (F = 2.270, *p* = 0.139) were found for lumbar lordosis.

For sacral slope, the analysis showed no significant interaction effect (F = 1.605, *p* = 0.212), no significant main effect of group (F = 1.312, *p* = 0.258), and no significant main effect of time (F = 1.385, *p* = 0.246).

### 3.2. Results of Trunk Symmetry

The results of trunk symmetry are presented in Table 5. A two-way ANOVA revealed no significant interaction effect (F = 1.207, *p* = 0.278) and no significant main effect of group (F = 0.029, *p* = 0.865). However, a significant main effect of time was observed for coronal balance (F = 37.460, *p* < 0.001). Post hoc comparisons for the main effect of time demonstrated a significant improvement in coronal balance following both the Rigo–WBV intervention (*p* < 0.001) and the Rigo–ONLY intervention (*p* = 0.005).

For scapular height asymmetry, the analysis showed no significant interaction effect (F = 1.760, *p* = 0.192) and no significant main effect of group (F = 3.075, *p* = 0.087), but a significant main effect of time was identified (F = 10.855, *p* = 0.002). Post hoc analysis indicated a significant within-group change in the Rigo–ONLY group (*p* = 0.010), with no evidence of a between-group difference in change (*p* > 0.05). Furthermore, no significant interaction effect (F = 0.329, *p* = 0.569), no significant main effect of group (F = 0.415, *p* = 0.523), and no significant main effect of time (F = 3.829, *p* = 0.057) were found for the distances of the scapulae from the spine.

### 3.3. Results of Angle of Trunk Rotation

The results of ATR are presented in Table 6. Two-way ANOVA revealed a significant interaction effect (F = 18.957, *p* < 0.001), no significant main effect of group (F = 3.541, *p* = 0.067), and a significant main effect of time (F = 20.765, *p* < 0.001) for ATR in the main thoracic curve. Post hoc comparisons for the main effect of time demonstrated a significant reduction in ATR in the main thoracic curve following the Rigo–WBV intervention (*p* < 0.001). ATR was significantly higher at baseline in the Rigo–WBV group (*p* = 0.032). However, after the intervention, ATR values were comparable between the two groups (*p* = 0.845).

For the thoracolumbar curve, no significant interaction effect (F = 0.030, *p* = 0.865) and no significant main effect of group (F = 1.090, *p* = 0.302) were observed, whereas a significant main effect of time was found (F = 49.850, *p* < 0.001). Post hoc analysis indicated a significant reduction in ATR following both the Rigo–WBV (*p* < 0.001) and Rigo–ONLY (*p* < 0.001) interventions.

In the lumbar curve, two-way ANOVA showed no significant main effect of group (F = 0.775, *p* = 0.384), but a significant interaction effect (F = 6.540, *p* = 0.014) and a significant main effect of time (F = 25.270, *p* < 0.001) were identified. Post hoc comparisons revealed a significant decrease in ATR in the lumbar curve following the Rigo–WBV intervention (*p* < 0.001).

Additionally, for ATR measured at the level of PSIS, no significant interaction effect (F = 2.996, *p* = 0.091) and no significant main effect of group (F = 2.981, *p* = 0.091) were found. However, a significant main effect of time was observed (F = 16.201, *p* = 0.002). Post hoc analysis demonstrated a significant reduction in ATR at the PSIS following the Rigo–WBV intervention (*p* < 0.001)

Due to the small number of proximal thoracic curves, statistical analysis for this curve was not performed.

## 4. Discussion

This study investigated the short-term clinical effects of incorporating WBV into the Rigo Concept approach on postural parameters in girls with AIS. The main findings included a reduction in ATR in the Rigo–WBV group and an improvement in coronal balance in girls in both study groups. In contrast, scapular height asymmetry decreased only in the Rigo–ONLY group. Apart from the Cobb angle, ATR represents an important clinical indicator for assessing the effectiveness of interventions in patients with AIS [2]. Notably, Fukuzawa et al. reported a strong correlation between ATR and the Cobb angle [28]. Therefore, a reduction in ATR may suggest a potentially beneficial effect of the applied intervention on radiological outcomes. However, it should be emphasized that over the 5-day intervention period, the absolute mean change in ATR in the Rigo–WBV group ranged from 1.0° to 2.1°, depending on the spinal segment assessed. Prowse et al. reported the standard error of measurement of the scoliometer to range from 1.23° to 1.5° [29]. Accordingly, the changes observed in the present study exceeded the measurement error for the main thoracic, thoracolumbar, and lumbar curves. Furthermore, in the Rigo–WBV group, effect sizes ranged from 0.51 to 0.95, indicating a medium effect for the main thoracic and thoracolumbar curves, as well as for the pelvis, and a large effect for the lumbar curve.

Improvement in aesthetics is one of the most important treatment goals for patients with AIS [3]. The significant improvement in coronal balance–and thus body aesthetics–observed in both the Rigo–WBV and Rigo–ONLY group highlights the value of this therapeutic approach. These findings are consistent with previous studies on PSSE effectiveness, which demonstrated superior outcomes compared with standard care, not including scoliosis-specific physiotherapy [2,3].

The experimental data suggest that scapular height is not reliably measurable, even by experts, as indicated by low ICC values. However, in healthy (symmetrical) participants, this value is very small, and measurement repeatability is likely to differ substantially in patients with scoliosis who present with marked asymmetry. Therefore, the clinical value of these parameters requires evaluation in subjects with scoliosis [23]. To the best of our knowledge, these parameters have not yet been defined for patients with scoliosis, despite being widely used in clinical practice.

Research published over the past five years has focused predominantly on the efficacy of Schroth therapy, likely due to its widespread use among physiotherapists and physicians and its structured methodology [2]. The Schroth method is historically linked to the Rigo Concept approach (Barcelona Scoliosis Physiotherapy School—BSPTS) [30]. Mohamed et al. reported that six-month Schroth physiotherapy added to standard care improved back asymmetry in patients with AIS [3]. These findings align with our findings, which demonstrate improvements in coronal balance. Notably, even short but intensive 5-day physiotherapy sessions appear capable of modifying postural parameters.

Lu et al. demonstrated that Schroth therapy combined with spiral stabilization improved both the Cobb angle and ATR in individuals with AIS [6]. Their intervention emphasized spinal elongation, core stabilization, and muscle coordination, with exercises focusing on vertical-axis training, muscle strength and balance, and symmetrical movement patterns [6]. In the present study, WBV was intended to enhance proprioception and increase trunk stabilization while maintaining specific 3D correction [10,11]. Vibration-assisted exercises, due to short training periods combined with a high number of muscular contractions, may improve muscle function [9]. This intensive stimulation appears particularly relevant for children with AIS, as participants receiving additional WBV demonstrated improvements in ATR across a greater number of spinal segments than the group undergoing the Rigo Concept alone.

In the available literature, only one study has investigated the use of WBV in the treatment of scoliosis. In that study, home-based physiotherapy using a vibration platform over six months was reported to be potentially useful in preventing curve progression [9]. However, the authors emphasized that WBV should be applied only as an adjunct to PSSE and not as a main therapy [9].

In the present study, the primary therapeutic approach was the Rigo Concept, which, through its classification system, allows individualized selection of exercise patterns tailored to scoliosis type [28]. This enables clinicians to provide optimal correction while maintaining the principle of individualized, patient-centered care. As a result, patients can be included in intervention groups performing exercises in the same starting positions, while still being appropriately adapted to each participant’s specific curve pattern. In a study by SharfEldin et al., comparing the Rigo Concept and Schroth method, both approaches produced positive therapeutic outcomes [4]. However, improvements in ATR and Cobb angle were more frequently observed in the Rigo Concept group [4]. Our findings confirmed that the Rigo Concept alone contributed to a reduction in ATR, but only in thoracolumbar curves. It is possible that Rigo Concept exercises based on derotational breathing are particularly effective in correcting this spinal region. The addition of WBV resulted in greater ATR reductions not only in this segment but also in the main thoracic and lumbar spine, as well as the pelvis.

The lack of improvement in scapular alignment following the Rigo Concept supplemented with WBV may be related to the short duration of the intervention or insufficient therapeutic influence on the muscle groups responsible for scapular positioning. Although improved spinal correction in the frontal and transverse planes was expected to positively affect scapular alignment, no such effect was observed. Similarly, no significant changes were found in sagittal spinal curvatures. Fang et al. [30] examined changes in sagittal curvatures in patients with AIS undergoing Schroth physiotherapy combined with bracing. Their intervention consisted of supervised Schroth exercises performed for 1.5 h per day, three times per week for six weeks, complemented by a daily 30–45-min home exercise program, and continued until skeletal maturity [30]. Such an intensive and long-term protocol allowed the authors to conclude that combining Cheneau bracing with Schroth therapy leads to better treatment outcomes, including improved correction of flatback and better quality of life in patients with AIS [30]. It is also possible that the Rigo Concept places a stronger emphasis on spinal derotation during expansion techniques and on achieving linearity in 3D correction, while comparatively less emphasis is placed on shaping sagittal-plane curvatures. However, this interpretation should be considered a post hoc hypothesis. The findings of this study provide a basis for future research on the effectiveness of the Rigo Concept alone and in combination with WBV. The observed improvements in clinical parameters such as ATR and coronal balance support the potential clinical value of supplementing PSSE with WBV. WBV represents a safe and easily applicable point for specific physiotherapy. Importantly, the present study does not allow evaluation of the isolated effects of WBV. Such an assessment would require randomization to WBV-only and no-treatment control groups, which poses ethical challenges in AIS management.

A limitation of the study is the short, five-day intervention period. Longer interventions would allow the assessment of long-term effects and inclusion of radiological outcomes, which remain the gold standard for AIS monitoring [28]. Nevertheless, the short protocol demonstrated that even brief but intensive physiotherapy sessions can influence clinical parameters. Additionally, the short duration ensured intervention consistency and allowed all sessions to be supervised by a single experienced physiotherapist. Consequently, the conclusions should be considered preliminary. However, they indicate a positive trend toward clinically meaningful changes, such as reductions in ATR and improvements in coronal balance. These outcomes may be particularly important for patients, as they directly influence body aesthetics. Changes observed over the 5-day intervention period should also be interpreted as postural changes of a neuromuscular nature. In AIS physiotherapy, modification of movement patterns and neuromuscular training often form the basis for subsequent structural changes. In contrast, the study by Langensiepen et al. [9] involved a six-month intervention combining supervised physiotherapy with home-based exercises performed under parental supervision or independently by the children. However, the quality and regularity of unsupervised home training remain uncertain, which may influence the reliability of long-term outcomes.

The lack of researcher blinding represents another limitation, as blinding was not feasible for organizational reasons. However, during the final measurement session, the researcher did not have access to the baseline results. Moreover, all assessments were performed according to predefined, standardized procedures to minimize potential assessment bias.

Among the strengths of this study is its focus on evaluating the effectiveness of the widely used Rigo Concept and its supplementation with WBV, which is not commonly applied in scoliosis therapy. On the one hand, this study contributes to the assessment of traditional PSSE effectiveness. On the other hand, it introduces a novel perspective on conservative scoliosis treatment. The limited number of comparable studies restricted direct comparison with other findings. Nevertheless, this work may contribute to future research on PSSE effectiveness.

## 5. Conclusions

The application of a five-day physiotherapy session based on the Rigo Concept, regardless of the use of WBV, does not affect the sagittal spinal curvatures in girls with mild-to-moderate AIS. Both the Rigo Concept alone and the Rigo Concept complemented with WBV performed in a standing position may improve coronal balance. Moreover, the Rigo Concept combined with WBV may have a positive effect on reducing ATR in girls with AIS. These findings reflect short-term clinical and postural changes and should not be interpreted as evidence of changes in Cobb angle or structural spinal correction. Further research is warranted to better understand the potential role of WBV as an adjunctive treatment in AIS. Continued investigation into the effectiveness of the Rigo Concept is also needed, particularly in light of its increasing clinical use and the need to support its application with robust scientific evidence.

## Figures and Tables

**Table 1:** Characteristics of participants (*n* = 45).

	Rigo–WBV *n* = 22	Rigo–ONLY *n* = 23	
	Mean ± SD	Mean ± SD	*p*
Age (years)	12.6 ± 1.9	12.9 ± 1.5	0.592
Body mass (kg)	48.0 ± 9.6	50.3 ± 10.5	0.578
Body height (m)	1.6 ± 0.1	1.6 ± 0.1	0.768
BMI (kg/m^2^)	18.4 ± 3.1	19.9 ± 2.6	0.153

Abbreviations: Rigo–WBV—Rigo Concept with whole-body vibration; Rigo–ONLY—Rigo Concept; BMI—body mass index.

**Table 2:** Radiological characteristics of the study group (*n* = 45).

	Rigo–WBV *n* = 22		Rigo–ONLY *n* = 23		
Cobb Angle	*n* Curves	Mean ± SD	*n* Curves	Mean ± SD	*p*
Proximal thoracic (°)	2	28.0 ± 2.8	2	26.0 ± 7.1	-
Main thoracic (°)	18	25.7 ± 7.4	20	24.3 ± 8.9	0.599
Thoracolumbar (°)	16	29.9 ± 8.9	15	25.3 ± 10.7	0.202
Lumbar (°)	7	18.4 ± 5.4	7	20.9 ± 6.8	0.475
Risser test		1.4 ± 2.0		1.6 ± 1.7	0.438

Abbreviations: Rigo–WBV—Rigo Concept with whole-body vibration; Rigo–ONLY—Rigo Concept.

**Table 3:** Sagittal curvatures of the spine, trunk symmetry, and angle of trunk rotation for Rigo–WBV and Rigo–ONLY before the intervention.

	Rigo–WBV *n* = 22	Rigo–ONLY *n* = 23	*p*
	Mean ± SD	Mean ± SD	
Sagittal curvature of the spine			
Thoracic kyphosis (°)	18.7 ± 12.7	24.4 ± 9.2	0.101
Upper thoracic kyphosis (°)	11.2 ± 7.6	12.0 ± 6.7	0.704
Lower thoracic kyphosis (°)	9.0 ± 8.7	11.9 ± 7.5	0.271
Lumbar lordosis (°)	29.6 ± 7.2	29.0 ± 9.3	0.800
Sacral slope (°)	24.0 ± 4.3	23.2 ± 4.9	0.549
Trunk symmetry			
Coronal balance (cm)	0.7 ± 0.6	0.6 ± 0.5	0.597
Scapular height asymmetry (°)	3.2 ± 1.9	4.3 ± 1.9	**0.035**
Distances of the scapulae from the spine (cm)	1.8 ± 1.3	2.0 ± 1.4	0.687
Angle of trunk rotation			
Proximal thoracic curve (°)	2.8 ± 1.5	1.9 ± 1.9	-
Main thoracic curve (°)	8.3 ± 4.0	5.7 ± 2.3	**0.009**
Thoracolumbar curve (°)	5.0 ± 3.4	5.9 ± 3.2	0.420
Lumbar curve (°)	3.9 ± 2.5	2.7 ± 2.5	0.120
Posterior superior iliac spine (°)	1.5 ± 1.5	0.8 ± 1.0	0.089

Abbreviations: Rigo–WBV—Rigo Concept with whole-body vibration; Rigo–ONLY—Rigo Concept; “-” due to the small number of curves, statistical analysis was not conducted; significant difference is bolded.

**Table 4:** Sagittal curvatures of the spine for the Rigo–WBV and Rigo–ONLY groups before and after 5-day physiotherapy.

Sagittal Curvature (°)	Rigo–WBV *n* = 22				Rigo–ONLY *n* = 23			
	Before	After			Before	After		
	Mean ± SD	Mean ± SD	Δ	ES	Mean ± SD	Mean ± SD	Δ	ES
Thoracic kyphosis	18.7 ± 12.7	19.8 ± 11.3	1.1	0.13	24.4 ± 9.2	25.9 ± 8.3	1.5	0.17
Upper thoracic kyphosis	11.2 ± 7.6	12.9 ± 8.0	1.7	0.22	12.0 ± 6.7	13.3 ± 5.4	1.3	0.21
Lower thoracic kyphosis	9.0 ± 8.7	8.6 ± 8.0	0.4	0.05	11.9 ± 7.5	11.1 ± 6.6	0.8	0.11
Lumbar lordosis	29.6 ± 7.2	31.1 ± 5.1	1.5	0.24	29.0 ± 9.3	29.6 ± 7.9	0.6	0.07
Sacral slope	24.0 ± 4.3	25.2 ± 5.0	1.2	0.26	23.2 ± 4.9	23.2 ± 3.8	0.0	0.00

Abbreviations: Rigo–WBV—Rigo Concept with whole-body vibration; Rigo–ONLY—Rigo Concept; Δ—absolute mean changes; ES—effect size.

**Table 5:** Trunk symmetry for the Rigo–WBV and Rigo–ONLY groups before and after 5-day physiotherapy.

	Rigo–WBV *n* = 22				Rigo–ONLY *n* = 23			
	Before	After			Before	After		
	Mean ± SD	Mean ± SD	Δ	ES	Mean ± SD	Mean ± SD	Δ	ES
Coronal balance (cm)	0.7 ± 0.6	0.2 ± 0.3	0.5	1.05	0.6 ± 0.5	0.2 ± 0.3	0.4	0.97
Scapular height asymmetry (°)	3.2 ± 1.9	2.7 ± 1.8	0.5	0.27	4.3 ± 1.9	3.2 ± 1.6	1.1	0.63
Distances of the scapulae from the spine (cm)	1.8 ± 1.3	1.5 ± 1.0	0.3	0.23	2.0 ± 1.4	1.8 ± 1.4	0.2	0.14

Abbreviations: Rigo–WBV—Rigo Concept with whole-body vibration; Rigo–ONLY—Rigo Concept; Δ—absolute mean changes; ES—effect size.

**Table 6:** Angle of trunk rotation for the Rigo–WBV and Rigo–ONLY groups before and after 5-day physiotherapy.

Angle of Trunk Rotation (°)	Rigo–WBV *n* = 22						Rigo–ONLY *n* = 23			
		Before	After				Before	After		
	*n* Curves	Mean ± SD	Mean ± SD	Δ	ES	*n* Curves	Mean ± SD	Mean ± SD	Δ	ES
Proximal thoracic curve	2	2.8 ± 1.5	1.7 ± 1.4	-		2	1.9 ± 1.9	1.2 ± 1.4	-	
Main thoracic curve	18	8.3 ± 4.0	6.4 ± 3.5	1.9	0.51	20	5.7 ± 2.3	5.7 ± 2.2	0.0	0.04
Thoracolumbar curve	16	5.0 ± 3.4	3.1 ± 2.6	1.9	0.63	15	5.9 ± 3.2	4.0 ± 2.4	1.9	0.67
Lumbar curve	7	3.9 ± 2.5	1.8 ± 1.9	2.1	0.95	7	2.7 ± 2.5	2.0 ± 2.0	0.7	0.31
PSIS	22	1.5 ± 1.5	0.5 ± 0.9	1.0	0.81	22	0.8 ± 1.0	0.3 ± 0.6	0.5	0.61

Abbreviations: Rigo–WBV—Rigo Concept with whole-body vibration; Rigo–ONLY—Rigo Concept; WBV—whole-body vibration; “-” due to the small number of curves, statistical analysis was not conducted; PSIS—posterior superior iliac spine; Δ—absolute mean changes; ES—effect size.

## Data Availability

The original data presented in the study are openly available in RepOD at https://doi.org/10.18150/OSMHLO (accessed on 19 December 2025).

## Abbreviations

The following abbreviations are used in this manuscript:

AIS	Adolescent idiopathic scoliosis
ATR	Angle of trunk rotation
BMI	Body mass index
BSPTS	Barcelona Scoliosis Physiotherapy School
PSIS	Posterior superior iliac spine
PSSE	Physiotherapeutic scoliosis-specific exercises
Rigo–ONLY	Rigo Concept group
Rigo–WBV	Rigo Concept with the whole-body vibration group
R1	Researcher 1
R2	Researcher 2
SOSORT	Society on Scoliosis Orthopaedic and Rehabilitation Treatment
WBV	Whole-body vibration
3D	three-dimensional
